# A Robust and Standardized Approach to Quantify Wound Closure Using the Scratch Assay

**DOI:** 10.3390/mps6050087

**Published:** 2023-09-17

**Authors:** Stefan Balko, Evan Kerr, Edward Buchel, Sarvesh Logsetty, Afshin Raouf

**Affiliations:** 1 Department of Immunology, Rady Faculty of Health Sciences, University of Manitoba, Winnipeg, MB R3E 0W2, Canada; stefan.balko@umanitoba.ca (S.B.); kerre3@myumanitoba.ca (E.K.); 2 CancerCare Manitoba Research Institute, CancerCare Manitoba, Winnipeg, MB R3E 0V9, Canada; 3 Max Rady College of Medicine, Rady Faculty of Health Sciences, University of Manitoba, Winnipeg, MB R3E 0W2, Canada; 4 Department of Surgery, Rady Faculty of Health Sciences, University of Manitoba, Winnipeg, MB R3E 0W2, Canada; edwardwayne.buchel@umanitoba.ca (E.B.); sarvesh.logsetty@umanitoba.ca (S.L.)

**Keywords:** wound healing, dermal fibroblasts, keratinocytes

## Abstract

The scratch assay is an in vitro assay that allows for high-throughput quantification of wound closure by keratinocytes and fibroblasts with relative ease. However, this assay is amenable to experimental variables, which can result in false-positive and false-negative data, making the interpretation of such data difficult. Also, data variability decreases the sensitivity of the scratch assay. Here, we identify important sources of data variation in the scratch assay and provide rational mitigation strategies that enable robust and highly reproducible quantification of scratch width and area, and ultimately the scratch closure rates. By eliminating these sources of variability, the sensitivity of the scratch assay is enhanced, thereby allowing for identification of dependent variables with wide-ranging impacts on wound closure in a robust and standardized manner.

## 1. Introduction

The repair of severe burns and other skin wounds is a complex process, which involves a cascade of highly orchestrated and regulated events that ultimately lead to the regeneration and remodeling of the injured skin. If the natural wound healing process is interrupted for any reason, chronic wounds occur, which are either healed through the formation of non-functional scar tissue, or progress into non-healing wounds [1,2,3]. Both of these undesirable outcomes lead to physical disability and emotional trauma that significantly impact patients’ quality of life [4]. The care and management of severe wounds are mostly supportive in nature, as the mechanisms that promote re-epithelialization and matrix deposition in the wounded area are not well understood. Several experimental approaches are currently used to investigate the different aspects of wound healing both in vivo and in vitro. In vivo animal models allow for the investigation of the complete wound healing process [5,6,7]. The in vitro models of wound healing include the use of synthetic skin equivalents and the scratch assay. Although skin equivalents are more anatomically representative, they pose complications with respect to imaging and image analysis. The scratch assay essentially allows for the quantification of the rate at which a scratch (gap) which is artificially introduced in a monolayer of fibroblasts or keratinocytes, closes. The relative ease with which the scratch assay can be set up allows for a high-throughput workflow, which is why this assay is frequently used to assess the impact of variables such as cytokines and growth factors on wound closure. However, the scratch assay is susceptible to variability in the results that can impact the interpretation of the data obtained. Here, we present steps that need to be considered to reduce variability in scratch assay data when using primary human dermal fibroblasts and keratinocytes. Such steps yield a robust and standardized approach to the scratch assay, allowing cross comparison of data obtained from different research teams, without requiring specialized equipment.

## 2. Experimental Design

The experiments used to generate the data discussed in this article are based on primary human dermal fibroblasts that were obtained from unused fat tissue samples. The primary human keratinocytes are from low-passaged primary adult human epithelial keratinocytes (HEKA) cells.

### 2.1. Materials

#### 2.1.1. Primary Human Dermal Fibroblast Cultures

Fibroblasts were obtained from the excess skin portion of abdominal fat flaps and were collected from patients undergoing breast reconstructive surgery. The fat grafts were collected based on written patient consent and with the approval of the University of Manitoba research Ethics Board (REB# HS24840). The excess skin samples were enzymatically and mechanically dissociated to yield primary dermal fibroblasts, as previously described [8]. Briefly, the skin was separated from adipose tissue and incubated with dispase overnight at 4 °C. Subsequently, the dermal layer was removed from the epidermal layer and incubated in collagenase (Type 1A from *Clostridium histolyticum*, Millipore Sigma, Cat # C9891) at 37 °C for 90 min with mechanical shaking. The dissociated dermal tissue was filtered through a 40 µm mesh filter, and the cells obtained were plated in a 10 cm tissue culture plate (passage 0 or P0), supplemented with fibroblast growth medium (DMEM/F12 containing 10% fetal bovine serum, FBS). Primary dermal fibroblasts were passaged when they reached 80% confluence using Acutase™ and were used at passages 3–5 (P3–5). All bio-specimens were obtained between July 2021 and December 2022, and all data for these experiments were collected and analyzed between January 2022 and April 2023. It is important to note that FBS could show batch to batch variation and therefore, we recommend testing different lots of FBS to ascertain which FBS batch provides consistent and optimum results.

#### 2.1.2. Primary Human Epidermal Keratinocyte–Adult (HEKa) Cultures

HEKa cells were obtained from ThermoFisher (ThermoFisher CAT#C005C) and placed in 10 cm tissue culture plates with keratinocyte growth medium 2 (PromCell Cat# C-20111) and kept in a humidified incubator at 37 °C with 5% CO_2_ with media changes every second day (P0). Upon 80% confluence, cells were passaged with Acutase™ and were only used up to passage 3 (P3). 

## 3. Procedure

### 3.1. Scratch Assay

HEKa cells (60,000), or primary dermal fibroblasts (10,000 cells) were placed in each well of a 24-well plate and were allowed to grow in keratinocyte or fibroblast growth media accordingly, until 90% confluent (usually within 3 days). At this point, the growth media was replaced with basal medium, containing no growth factors, FBS or supplements (PromoCell CAT# 20211 for keratinocytes or DMEM/F12 for fibroblasts) for 18–24 h to synchronize cells at the quiescence stage (G_0_) of cell cycle. Subsequently, a full-thickness scratch was introduced in the middle of the cell monolayer with a 200 µL or 1000 µL pipette tip as indicated, and the plates were washed several times with PBS to remove cell debris. PBS was added to each well and digital images were obtained to quantify the initial scratch width and area (0 h) using an inverted microscope. The HEKa cells were then supplemented with keratinocyte basal medium (5 µg/mL insulin, 0.33 µg/mL hydrocortisone, 10 µg/mL transferrin, 0.125 ng/mL EGF and 0.06 mM CaCl_2,_ all final concentrations)_,_ and the fibroblasts were supplemented with DMEM/F12 containing 0.5% fetal bovine serum. This was carried out to provide cells with the minimum concentration of growth factors needed for survival and proliferation, thereby facilitating the study of subtle changes in scratch (i.e., wound) closure resulting from the experimental variables. For the purpose of quantifying wound closure rates, digital images were obtained at 0 h and 12 h, 24 h or 36 h, as indicated. The extent of wound closure was determined by comparing percent wound closure to the initial (0 h) scratched area. The optimal seeding densities for the HEKa and primary fibroblasts were determined experimentally.

### 3.2. Image Analysis

Images were taken using an Evos M5000 (Thermo Fisher Scientific, Waltham, MA, USA) inverted microscope at 4× and 10× magnifications. Images were analyzed using an ImageJ macro developed in our laboratory (Supplementary Materials), which is specifically designed to measure scratch area, subtract any cells that may have migrated into the scratched area, and output the final scratch area in pixels. The ImageJ measurement tool was used to measure the scratch widths with measurements taken at the center of the scratch in each picture, from wall to wall. The percentage of the wound (i.e., scratched area) that remained open was obtained by dividing the scratch area at 12 h, 24 h, and 36 h time points by the scratch area at 0 h time point.

## 4. Expected Results

### 4.1. Scratch Width Is a Major Factor in Scratch Closure Rate

**Figure 1:**
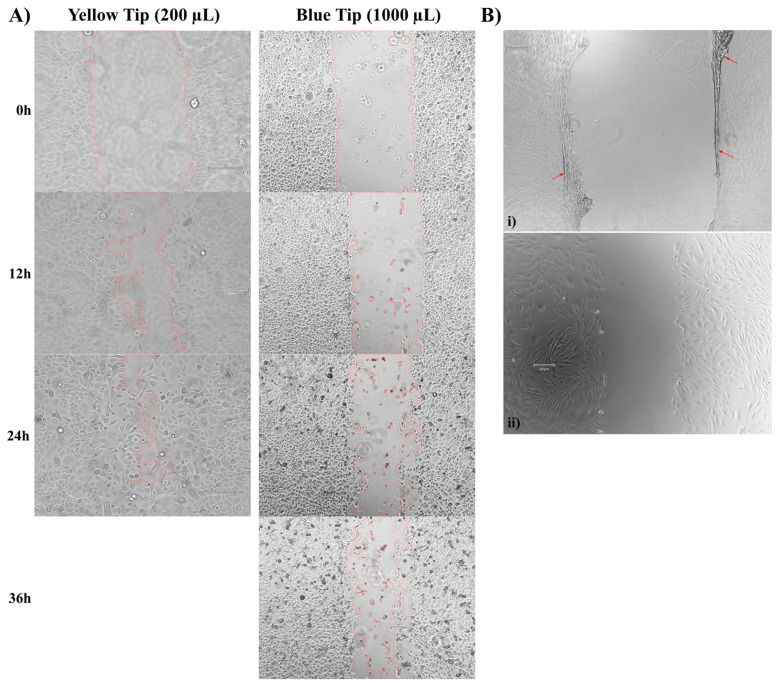
Scratch width and seeding density plays a major role in creating consistent and reproducible scratches. (**A**) Representative wound outline images of HEKa cells scratched with yellow pipette tips or blue pipette tips, over a 36 hr period. Yellow tip images were taken at 10× magnification; blue tip images were taken at 4× magnification. (**B**) Representative images of scratches created in primary dermal fibroblasts at different seeding densities; (**i**) 60,000 cells seeded in a single well of a 24-well plate, (**ii**) 10,000 cells seeded in a single well of a 24-well plate. All cells were allowed to incubate for 3 days before scratching. Red arrows are highlighting cell sheet layer folding over itself. Images were taken at 4× magnification.

To examine the impact of dependent variables on wound closure, the scratch assay needs to have high sensitivity to detect a dynamic range of changes to the scratched area. In our experience, however, scratches with smaller widths tend to close in under 24 h and did not allow accurate quantification of the impact of dependent variables on the rate of wound closure. We therefore compared the width of scratches introduced into a monolayer of human epidermal keratinocyte-adult (HEKa) cells utilizing the commonly used 200 µL and the 1000 µL pipette tips (Table S1). We found that the 1000µL pipette tips, on average, created a wider scratch area, with an average width of 1104 ± 152 µm, compared to an average width of 795 ± 96 µm created from the 200 µL pipette tips. These wider scratches closed in 36 h compared to the scratches made by the 200 µL pipette tips, which closed in 24 h (Figure 1A). Based on this result, 1000 µL pipette tips were used to create the initial scratches.

Because the rate at which the scratched areas are closed is heavily influenced by cell proliferation and/or migration, we hypothesized that cell density could be an important modulator of wound closure rates in the scratch assay. To this end, we set up scratch assays using either primary human dermal fibroblasts or HEKa cells with different cell densities. In the case of HEKa cells, we found that placing 50,000 to 60,000 cells per each well of a 24-well plate was sufficient to allow the cultures to reach 90% confluency in 3 days. However, at higher seeding densities (>60,000 cells/well), HEKa cells contained a higher frequency of terminally differentiated cell phenotype (larger, flat cell). Such terminally differentiated cells are typically observed in HEKa cells at higher passages (>P5) that can no longer be maintained in culture [9]. We also noticed that, at higher densities (>50,000 cells/well), the primary dermal fibroblasts formed a contiguous sheet of cells after 3 days, causing the cells to rip and fold as opposed to creating a clean scratch (Figure 1B(i). However, at lower densities (10,000 cells/well), this sheet did not form, allowing for consistent clean scratches (Figure 1B(ii). Based on these observations, we have determined that 5263 cells/cm^2^ for primary low-passage human dermal fibroblasts and 31,578 cells/cm^2^ for HEKa cells are appropriate cell densities to use when setting up monolayer scratch assays. We also observed that the primary dermal fibroblasts are sensitive to prolonged exposure to PBS (>20 min), but this issue can be mitigated by using Hank’s balanced salt solution (HBSS) instead. Use of PBS to cover the HEKa cells did not alter their cell morphology and they remained attached to the plates.

### 4.2. Quantifying Total Scratch Area Is Essential for Obtaining Consistent Wound Closure Rates

**Figure 2:**
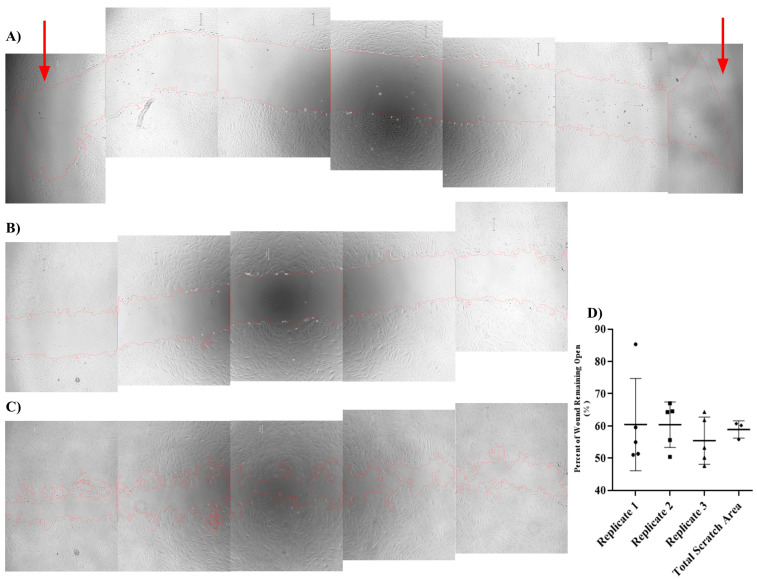
Scratch wound closure measurement variability is drastically reduced when imaging 76% of the entire length of the scratch. (**A**) Representative image of all 7 fields of view, with arrows highlighting both the first and last view, where the scratch becomes inconsistent and does not carry through the entire frame. (**B**,**C**) Representative dermal fibroblast scratches showing 5 fields of view, taken sequentially, to encapsulate 76% of the scratch area, at 0 h (**B**) and 12 h (**C**), showing the variability in the scratch closure observed in each frame. Images were taken at 4× magnification. (**D**) Scatter plot of scratch wound closure over 12 h period, represented as percent of wound remaining open. Each technical replicate is based on 5 different, single fields of view of the scratched area. All 5 fields of view from each technical replicate were then added together, and the average of all 3 technical replicate summations was plotted. Data are represented as Mean ± SD.

Typically, scratch closure rate is determined by measuring the scratch width from a single field of view of a representative scratched area over a given period of time. However, we found that relying on a single field of view resulted in highly variable scratch area data, making it difficult to obtain reproducible results. We hypothesized that quantifying the majority of the length of scratches in each well would produce more consistent results and allow for more accurate quantification of the scratch area and wound closure rates. To this end, we set up scratch assays using our optimized conditions and determined that 5 digital images, taken sequentially at 4× magnification, were sufficient to cover, on average, 76 ± 2.6% of the scratched length in each well of a 24-well plate (Figure 2A). The images from the top and the bottom of the well were omitted from the analysis since these images contained the blunt ends of the scratches and were highly variable and inconsistent (Table S2). The total scratch area for each of the 3 technical replicates was obtained by combining the scratch areas of the 5 fields of view taken per each replicate. This was then compared to the average scratch area of the 5 fields of view per replicate scratch (Figure 2B–D). Although the average scratched area per field of view showed great variability, the total scratch area per replicate produced much more robust data. (Table S3 and Figure 2D).

## 5. Discussion

The scratch assay is a convenient in vitro technique that offers a controlled and yet malleable assay to examine the impact of different experimental conditions on the wound closure properties of keratinocytes and fibroblasts [9,10]. To this end, HEKa cells and primary human dermal fibroblasts are frequently used. However, the scratch assay is amenable to large variability in the scratch closure data that could result in false positive or false negative results.

Here, we have identified critical sources of variability in the scratch assay and provide rational approaches to mitigate them. Typically, smaller 200 µL pipette tips are used to generate scratches [10,11,12] but we found that the use of small pipette tips creates narrower scratches and results in a faster scratch closure rate, which makes the assessment of dependable variables with medium impact on wound closure very challenging. We also found that initial seeding density is important for ensuring consistency in the initial scratched area. This issue was particularly important when using primary human dermal fibroblast, where higher seeding densities resulted in the formation of a uniform sheet of cell, leading to less control over the initial scratch widths. To assess wound closure rates in scratch assays, typically, a representative view of the scratched area is quantified over time [10,12], or a set of images over a smaller segment of the scratch [9] is considered. Here, we show that it is essential to consider the full length of each scratch to obtain an accurate representation of wound closure. The number of pictures needed to cover the length of the scratch will depend on the tissue culture well size, but at least 76% of the scratched area needs to be covered. In this way, the size of tissue culture plates used for scratch assays will not impact the wound closure rates. In our work, obtaining lower magnification pictures decreases the number of pictures needed to cover the length of a scratch. However, these images lack the resolution needed for optimal quantification of the scratch area and lead to data variability. Many software options are available to quantify the width of scratches that are documented in digital photographs. We found that such software do not sufficiently address the migration of cells into the wounded area. The ImageJ macro that we developed (Supplementary Materials), accurately outlines the wound edges, and identifies any cells migrated into the scratched area, allowing it to generate a more accurate measurement of the wound closure. This macro also allows the user to make manual adjustments to improve identification of the leading edge of the scratched area. Using the ImageJ software, which is available to the research community at no charge, and our macro, it is possible to stitch all images from each replicate scratch together to obtain the total scratch area (Figure 2), thus reducing the image analysis workload.

## 6. Conclusions

Based on our data, we suggest the HEKa cells should be seeded at 31,578 cells/cm^2^ and primary human fibroblasts should be seeded at 5263 cells/cm^2^ for the purpose of creating a monolayer of cells that can be used in the scratch assay. Moreover, at the least 76% of the scratched area should be documented to allow accurate and robust measurements of wound closure rate. By eliminating data variability, the scratch assay can be used as an excellent approach to study factors that have a significant impact on wound closure. Adherence to a standardized scratch assay protocol such as the one described here allows for the cross comparative analysis of data generated by different research teams, which can then be compiled to create large datasets of cytokines, chemokines, and growth factor responses on wound closure using the scratch assay.

## Data Availability

The data presented in this study are available in Supplementary Materials.

## Supplementary Materials

The following supporting information can be downloaded at: https://www.mdpi.com/article/10.3390/mps6050087/s1, Supplementary File S1: Scratch Outline Macro ImageJ; Table S1. Average width of scratches created in HEKa monolayer cells, using a yellow pipette tip and blue pipette tip; Table S2: Total area of scratch measured in each sample well, measured in pixels. Rows highlighted in red indicate fields of view emitted from the total scratch area used for calculation; Table S3: Scratch wound area, measured over 12 h period, of all 5 fields of view taken per well, with the summation of each field of view also. Area represented as pixels as well as percent of the original scratch.
