# Supplementary material for: A Robust and Standardized Approach to Quantify Wound Closure Using the Scratch Assay
Source: Methods Protoc. 2023 Sep 17;6(5):87. doi: 10.3390/mps6050087 (PMC10514857; doi:10.3390/mps6050087)
Supplement: Supplementary file 1 [file mps-06-00087-s001.zip › mps-2551077-supplementary.pdf]

Table S1. Average width of scratches created in HEKa monolayer cells, using a yellow pipette tip and blue pipette tip.

Yellow Tip	Width (μm)	Blue Tip	Width (μm)
Sample 1 a)	713.35	Sample 1 a)	914.414
b)	774.687	b)	959.396
c)	809.949	c)	1316.972
Sample 2 a)	712.191	Sample 2 a)	1230.454
b)	733.82	b)	1211.442
c)	717.102	c)	1204.288
Sample 3 a)	904.736	Sample 3 a)	985.767
b)	795.088	b)	1011.08
c)	990.144		
Avg. Width	794.563		1104.227
SD	96.23714		152.3225

Note: a, b and c refer to different technical replicates within each sample

Table S2. Total area of scratch measured in each sample well, measured in pixels. Rows highlighted in red indicate fields of view emitted from the total scratch area used for calculation.

	Sample 1 (pixels)	Sample 2 (pixels)	Sample 3 (pixels)
Field of View 1	781,068	820,421	805,117
Field of View 2	1,106,869	1,218,963	1,189,862
Field of View 3	1,185,137	1,242,776	1,134,209
Field of View 4	1,233,318	1,278,831	1,077,086
Field of View 5	1,244,192	1,258,116	1,275,993
Field of View 6	1,194,878	1,289,073	1,525,943
Field of View 7	1,173,379	773,420	1,245,388
Total	7,918,841	7,881,600	8,253,598
Total Scratch Area %	75.32	79.78	75.16
Average of Total Scratch Area	76.75		
SD	2.62		

Table S3. Scratch wound area, measured over 12-hr period, of all 5 fields of view taken per well, with the summation of each field of view also. Area represented as pixels as well as percent of the original scratch.

	0 h area	12 h area	0 h area (%)	12 h area (%)
Replicate 1-1	888,480.0	528,908.0	100.0	59.5
Replicate 1-2	930,143.0	793,298.0	100.0	85.3
Replicate 1-3	938,988.0	515,935.0	100.0	54.9
Replicate 1-4	958,758.0	488,960.0	100.0	51.0
Replicate 1-5	1,027,254.0	527,436.0	100.0	51.3
Replicate 1 total	4,743,623.0	2,854,537.0	100.0	60.2
Replicate 2-1	794,649.0	4004,10.0	100.0	50.4
Replicate 2-2	845,893.0	470,299.0	100.0	55.6
Replicate 2-3	896,683.0	600,990.0	100.0	67.0
Replicate 2-4	871,222.0	562,381.0	100.0	64.6
Replicate 2-5	959,130.0	616,198.0	100.0	64.2
Replicate 2 total	4,367,577.0	2,650,278.0	100.0	60.7
Replicate 3-1	1,033,879.0	491,802.0	100.0	47.6

Replicate 3-2	1,103,281.0	553,175.0	100.0	50.1
Replicate 3-3	1,100,017.0	585,185.0	100.0	53.2
Replicate 3-4	1,136,240.0	701,781.0	100.0	61.8
Replicate 3-5	1,266,780.0	815,026.0	100.0	64.3
Replicate 3 total	5,640,197.0	3,146,969.0	100.0	55.8
Replicate avg	4,917,132.3	2,883,928.0	100.0	58.9
Replicate STD	653,811.5	249,646.5	0.0	2.7
